# Spotlight on Ki67 as a prognostic marker in early breast cancer: all that glitters may not be gold

**DOI:** 10.1186/s13000-020-01024-9

**Published:** 2020-09-11

**Authors:** Roberta Maltoni, Michela Palleschi, Sara Ravaioli, Maria Maddalena Tumedei, Mattia Altini, Sara Bravaccini

**Affiliations:** Istituto Scientifico Romagnolo per lo Studio e la Cura dei Tumori (IRST) IRCCS, Via P. Maroncelli 40, 47014 Meldola (FC), Italy

**Keywords:** Ki67, PgR, Breast cancer

## Abstract

Kang and colleagues evaluated on a case series of 1848 breast cancer (BC) patients operated for a first primary ER positive HER2 negative BC if Ki67 expression is a significant prognostic factor only when PgR expression is low. The authors concluded that Ki67 with 10% cut off value is a prognostic factor only under low PgR expression level in early BC. We would like to underline, that as already stated in our previous papers, we believe that proliferation is important to define the decision-making of adjuvant therapies in early BC. The issue on Ki67 detection is the poor reproducibility due to different antibody clones, platforms and scoring methods. Not less important is that different Ki67 cut off values have been used by San Gallen guidelines and changed overtime. Then, despite the interesting results, we believe it would be better to use Ki67 biomarker in according to the standard Ki67 cut off according to San Gallen guidelines. Nowadays, standardization and optimization of Ki67 is still an urgent need.

Ki67 value has been recognized in early breast cancer (BC) but controversy still exists. We congratulate with Kang and colleagues for the very interesting paper. The authors evaluated in 1848 BC patients operated on for a first primary Estrogen Receptor (ER) positive HER2 negative BC if Ki67 expression is a significant prognostic factor only when Progesterone Receptor (PgR) expression is low [1]. The authors concluded that Ki67 with 10% cut off value is a prognostic factor only under low PgR expression level in early BC. PgR should be considered in evaluating the prognosis of BC patients using Ki67 expression.

About 30 years ago, our group started a study in attempt to establish the use of chemotherapy or nothing in node negative BC with high tumor labelling index [2]. In 2008, our research group published the results from this phase 3 randomized multicenter study on the effects of adjuvant Cyclophosphamide, Methotrexate, Fluorouracil (CMF) in patients with node-negative, rapidly proliferating BC [2]. The results from twelve-year subgroup analysis showed that CMF produced a 25 and 20% relative reduction in relapse and death cumulative incidence, respectively.

We recently demonstrated that PgR is an independent prognostic marker in rapidly proliferating hormone receptor positive early BC [3]. We would like to underline that, as already stated in our previous papers, we believe that proliferation is important to define the decision-making of adjuvant therapies in early BC [2].

The issue on Ki67 detection is the poor reproducibility due to different antibody clones, platforms and scoring methods used among the different laboratories and observers as reported by the studies on biomarkers comparison among the different centers [4]. As we already observed in our laboratory its detection by immunohistochemistry is affected by the preanalytical phase in terms of tissue over and under-fixation or delay in fixation (Fig. 1) [5]. All this factors affect reproducibility of the different studies [4]. Not less important is that different Ki67 cut off values have been used by San Gallen guidelines and changed overtime from 14 to 30%. Then, despite the interesting results, we believe it would be better to use Ki67 biomarker in according to the standard Ki67 cut off according to San Gallen guidelines. Nowadays, standardization and optimization of Ki67 is still a utopia and further efforts are required.

**Fig. 1:**
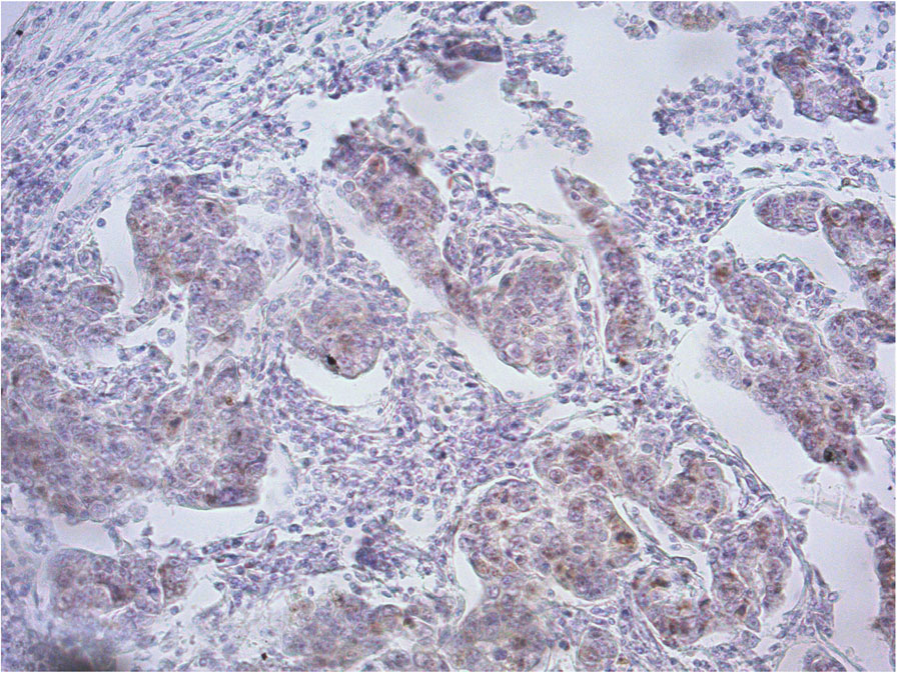
Invasive breast cancer tissue with non-specific and inhomogeneous expression of Ki67 by immunohistochemistry (20X magnification)

## Data Availability

Data sharing is not applicable to this article as no datasets were generated or analyzed during the current study.

## Abbreviations

BC: Breast cancer
ER: Estrogen Receptor
PgR: Progesterone Receptor
CMF: Cyclophosphamide, Methotrexate, Fluorouracil

## References

[1] Kang YJ, Lee HB, Kim YG, Han J, Kim Y, Yoo TK, et al. Ki-67 expression is a significant prognostic factor only when progesterone receptor expression is low in estrogen receptor-positive and HER2-negative early breast Cancer. J Oncol. 2019;7386734. 10.1155/2019/7386734 PMC6949686 31975992

[2] Amadori D, Nanni O, Volpi A, Casadei Giunchi D, Marangolo M, Livi L (2008). Phase III randomized multicenter study on the effects of adjuvant CMF in patients with node-negative, rapidly proliferating breast cancer: twelve-year results and retrospective subgroup analysis. Breast Cancer Res Treat.

[3] Bravaccini S, Bronte G, Scarpi E, Ravaioli S, Maltoni R, Mangia A (2020). The impact of progesterone receptor expression on prognosis of patients with rapidly proliferating, hormone receptor-positive early breast cancer: a post hoc analysis of the IBIS 3 trial. Ther Adv Med Oncol.

[4] Polley MY, Leung SC, Gao D, Mastropasqua MG, Zabaglo LA, Bartlett JM (2015). An international study to increase concordance in Ki67 scoring. Mod Pathol.

[5] Amadori D, Serra P, Bravaccini S, Farolfi A, Puccetti M, Carretta E (2014). Differences in biological features of breast cancer between Caucasian (Italian) and African (Tanzanian) populations. Breast Cancer Res Treat.

